# Reversible Zn Metal Anodes Enabled by Trace Amounts of Underpotential Deposition Initiators

**DOI:** 10.1002/anie.202301192

**Published:** 2023-03-24

**Authors:** Yuhang Dai, Chengyi Zhang, Wei Zhang, Lianmeng Cui, Chumei Ye, Xufeng Hong, Jinghao Li, Ruwei Chen, Wei Zong, Xuan Gao, Jiexin Zhu, Peie Jiang, Qinyou An, Dan J. L. Brett, Ivan P. Parkin, Guanjie He, Liqiang Mai

**Affiliations:** ^1^ State Key Laboratory of Advanced Technology for Materials Synthesis and Processing Wuhan University of Technology Wuhan 430070 P. R. China; ^2^ Christopher Ingold Laboratory Department of Chemistry University College London London WC1H 0AJ UK; ^3^ Electrochemical Innovation Lab Department of Chemical Engineering University College London London WC1E 7JE UK; ^4^ Institute of Technological Sciences Wuhan University Wuhan 430072 P. R. China; ^5^ Department of Materials Science and Metallurgy University of Cambridge Cambridge CB3 0FS UK; ^6^ Beijing Key Laboratory of Theory and Technology for Advanced Batteries Materials School of Materials Science and Engineering Peking University Beijing 100871 P. R. China

**Keywords:** Aqueous Battery, Electrolyte Additive, Interfacial Electrochemistry, Underpotential Deposition, Zinc Anode

## Abstract

Routine electrolyte additives are not effective enough for uniform zinc (Zn) deposition, because they are hard to proactively guide atomic‐level Zn deposition. Here, based on underpotential deposition (UPD), we propose an “escort effect” of electrolyte additives for uniform Zn deposition at the atomic level. With nickel ion (Ni^2+^) additives, we found that metallic Ni deposits preferentially and triggers the UPD of Zn on Ni. This facilitates firm nucleation and uniform growth of Zn while suppressing side reactions. Besides, Ni dissolves back into the electrolyte after Zn stripping with no influence on interfacial charge transfer resistance. Consequently, the optimized cell operates for over 900 h at 1 mA cm^−2^ (more than 4 times longer than the blank one). Moreover, the universality of “escort effect” is identified by using Cr^3+^ and Co^2+^ additives. This work would inspire a wide range of atomic‐level principles by controlling interfacial electrochemistry for various metal batteries.

## Graphical abstract

M is a metal with a higher reduction potential and work function than Zn. If M^n+^ is used as an electrolyte additive, M^n+^ will deposit preferentially and then render UPD of Zn uniformly on M. Moreover, the deposited M can dissolve back into the electrolyte after Zn stripping and thus does not increase the interfacial charge transfer resistance, enabling a highly reversible Zn anode. Ni^2+^, Cr^2+^, and Co^2+^ have been verified to function as M^n+^.

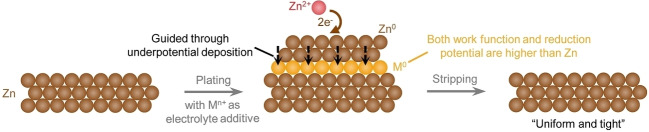

## Introduction

Metallic zinc (Zn) has a relatively low potential (−0.76 V vs. standard hydrogen electrode (SHE)) among the metals that can be stably presented in aqueous electrolytes, exhibiting a high output voltage when used as the anode.^[1]^ Moreover, it possesses a high theoretical capacity (820 mAh g^−1^), abundant sources, low cost, and non‐toxicity. Thus, Zn has been regarded as an ideal anode material for aqueous batteries.^[2]^ However, the cyclic reversibility of Zn anodes is still insufficient, especially at low current densities.^[3]^ There is an urgent need to address the severe Zn dendrite and hydrogen evolution reaction (HER) issues to advance the industrialization of aqueous zinc batteries.^[4]^

Previous research focused on constructing coatings on Zn anodes or introducing electrolyte additives.^[5]^ The former inevitably increases the interfacial charge transfer resistance and, in the meantime, fails once the coating cracks or detaches.^[6]^ In contrast, electrolyte additives are self‐healing.^[7]^ Namely, they improve the cyclic reversibility of Zn anodes by conformally covering the electrode surface. Generally, electrolyte additives function in two ways, one relies on the in situ generation of a protective solid‐electrolyte interphase^[8]^ (SEI, which is thinner and more compatible than artificial coatings), but the in situ formed SEI consumes electrolytes irreversibly and still inevitably increases the interfacial charge transfer resistance.^[9]^ The other way utilizes the properties of functional groups, such as shielding^[10]^ or hydrophobic^[11]^ effects. However, these effects act in an ionic form and fundamentally have a limited guiding effect. This is because there are various ions in the electrolyte and the targeted ions would not cover the entire surface of the electrode.^[12]^ A method that combines the advantages of coatings and additives while avoiding their drawbacks is still undeveloped.

Underpotential deposition (UPD) is a phenomenon of electrodeposition of species at a potential higher than the equilibrium one. It occurs when the substrate (A) has a higher work function than the deposited metal (B), making the A‐B interaction stronger than the B‐B interaction.^[13]^ Thus, UPD can be understood as when a metal can be deposited on another material more easily than it can be deposited on itself.^[14]^

**Scheme 1:**
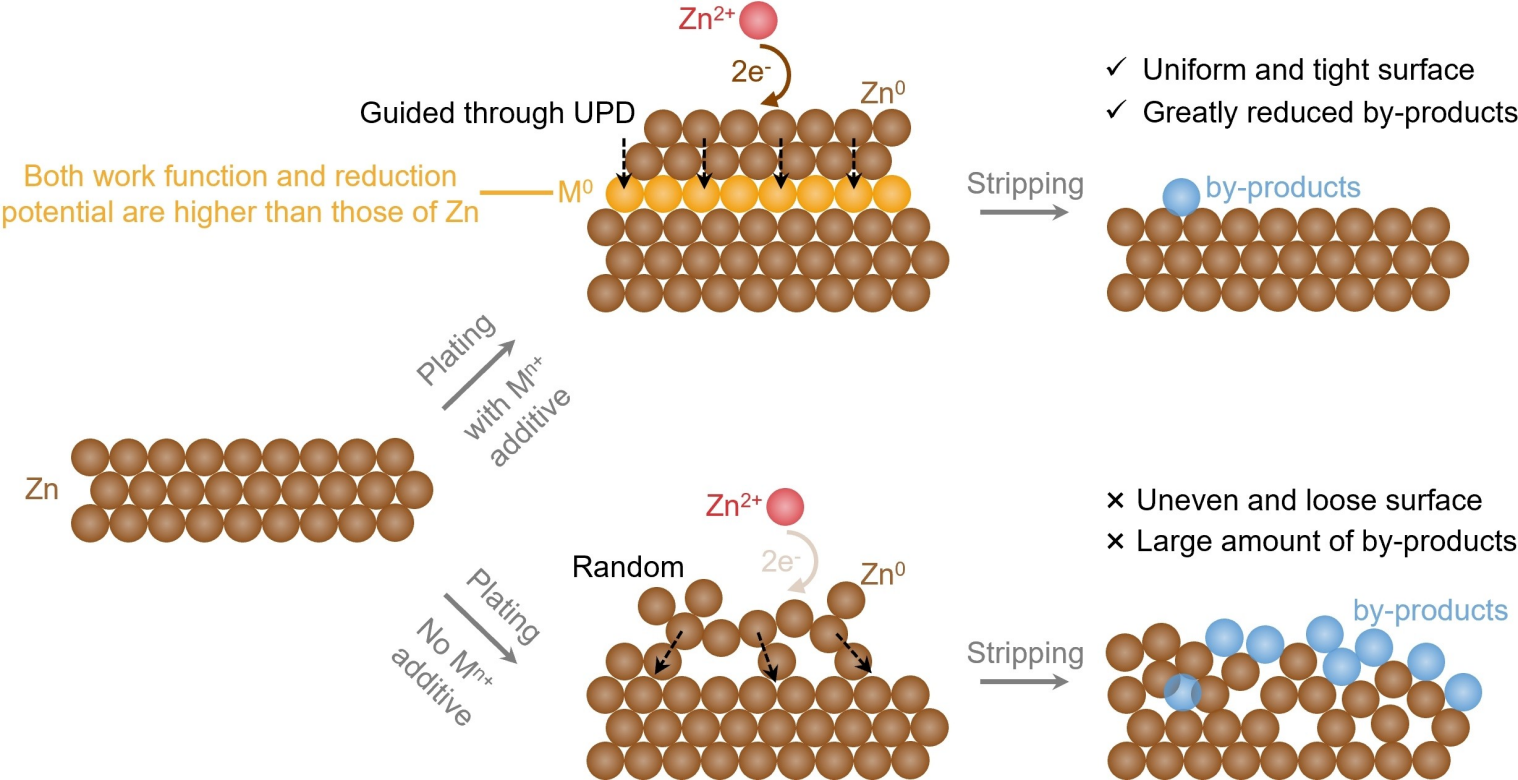
Schematic representation of the “escort effect” of the electrolyte additive, M^n+^, based on the underpotential deposition (UPD) mechanism.

Here, we propose an “escort effect” of electrolyte additives based on the UPD mechanism. Taking Ni^2+^ additive as an example, such a metal with higher work function (5.15 eV) and reduction potential (−0.257 V vs. SHE) than Zn (4.33 eV and −0.76 V vs. SHE) would preferentially deposit and enable UPD of Zn on Ni, thus facilitating nucleation and growth of Zn uniformly, exhibiting smooth Zn surface without apparent dendrite or by‐product formation. Subsequently, Ni would dissolve after Zn stripping such that no extra interfacial charge transfer resistance is induced (Scheme 1). Combining theoretical calculations and characterizations, including distribution of relaxation times (DRT) analysis towards in situ electrochemical impedance spectroscopy (EIS), the “escort effect” of Ni^2+^ was demonstrated. In addition, the “escort effect” was also verified in the system utilizing Cr^3+^ or Co^2+^ additives. This design idea based on atomic‐level interfacial electrochemistry provides an effective strategy to develop highly reversible metal anodes.

## Results and Discussion

**Figure 1:**
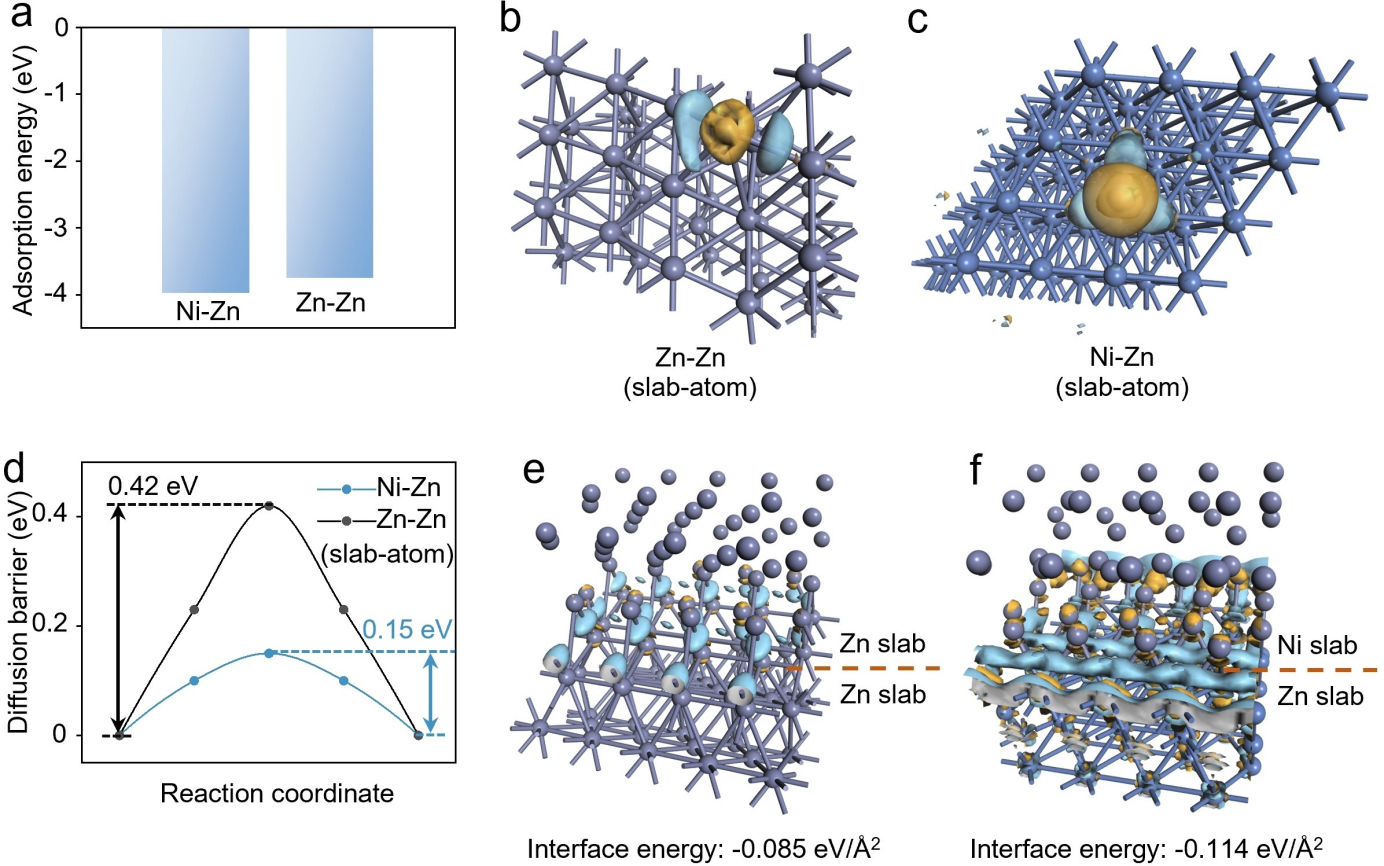
Theoretical calculations based on the DFT method. a) Binding energy of Zn atoms adsorption on the bare Zn and Ni surfaces. Calculated charge density difference for Zn atoms adsorbed on the b) Zn and c) Ni surfaces. The yellow and blue surfaces correspond to the gain and loss of charge, respectively. d) Energy for diffusion of Zn^2+^ on Ni and Zn surfaces. The calculated charge density difference between e) Zn slabs and Zn slabs and f) Zn slabs and Ni slabs. The stick at the upper part is hidden for a better view of the interface.

The first‐principles calculations based on the density functional theory (DFT) method were first conducted to investigate the underlying mechanism of the “escort effect”. At first, the adsorption energy of the Zn atom on Zn and Ni slabs are calculated to be −3.75 eV and −3.98 eV, respectively (Figure 1a), exhibiting a stronger affinity between the Zn atom and the Ni slab than that between the Zn atom and the Zn slab. This indicates that the deposition of (sub)monolayer Zn atoms on Ni is easier than on Zn. The charge density difference plot of the Zn atom on different slabs (Ni and Zn) further pictures the stronger chemical bond formed between the Zn atom and the Ni slab (Figure 1b,c), which suggests a more accessible nucleation of Zn on Ni. Moreover, the transition barrier when the Zn atom transfer on the Ni or Zn slab helps to illustrate the above results. In addition, the subsequent growth of Zn metals after nucleation was investigated. The transition barrier of the Zn atoms on the Ni and Zn slabs are 0.15 and 0.42 eV, individually (Figure 1d). It confirms that the migration of Zn atoms on the surface of Ni slabs can be much faster than that on Zn slabs. Beyond Zn atoms, we also studied the affinity of Zn slabs with Ni and Zn slabs by performing the charge density difference (Figure 1e,f). Apparently, more transferred electrons between the Ni−Zn slabs were observed than on the Zn−Zn slabs, while the interface energy of Ni−Zn slabs (−0.114 eV Å^−2^) is lower than Zn−Zn slabs (−0.085 eV Å^−2^). This illustrates a denser growth of the Zn metal on the Ni substrate than on the Zn substrate. These calculations indicate that Zn is more prone to nucleation and growth on Ni substrates than on Zn substrates.

**Figure 2:**
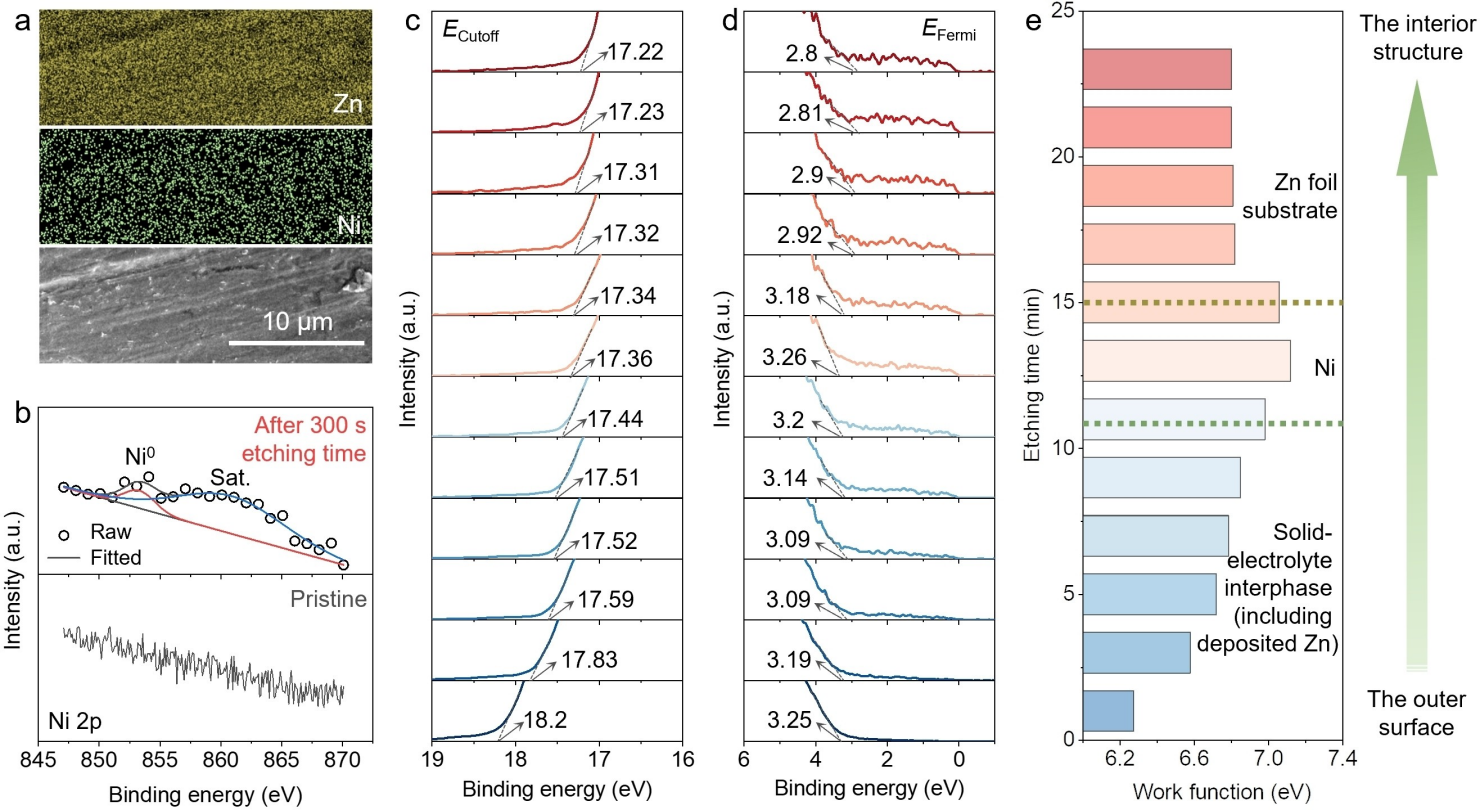
Structural characterizations of the Zn electrode in a Zn||Zn symmetric cell with a Ni^2+^‐ZS electrolyte after 1 h plating at 1 mA cm^−2^. a) SEM and corresponding EDS mappings. b) Ni 2p XPS spectra at the pristine state and after 300 s etching time. Detailed investigation of the Zn surface after 4 min plating at 1 mA cm^−2^, including c), d) UPS spectra at states after etching for 1 min, 3 min, 5 min, 7 min, 9 min, 11 min, 13 min, 15 min, 17 min, 19 min, 21 min, and 23 min from bottom to top, and e) Calculated work function at corresponding states in c) and d).

Based on the theoretical calculations, we conducted experimental investigations. After the Zn||Zn symmetric cell cycled with the 2 M ZnSO_4_+0.004 M NiSO_4_ electrolyte (denoted as Ni^2+^‐ZS, while bare ZnSO_4_ is denoted as ZS), abundant signals of Zn and Ni on the surface of the zinc foil can be seen in the scanning electron microscope (SEM) image and corresponding energy dispersive spectrometer (EDS) mapping images (Figure 2a). Moreover, from the X‐ray photoelectron spectroscopy (XPS) spectra, the signal of Ni is absent on the surface while occurring after 300 s etching time (Figures 2b and S1). More importantly, the surface of electrodes at different depths (after etching at different times) was probed by ultraviolet photoelectron spectroscopy (UPS). From Figure 2c–e, the work function of the surface increases first and then decreases with the increasing etching time (while the one cycled with ZS did not generate such evolution of the work function, Figure S2), which corresponds to the configuration of solid‐electrolyte interphase (including the deposited Zn), deposited Ni, and Zn foil substrate from the surface to the bulk. Then the Ni signal disappeared after stripping for 1 h at 1 mA cm^−2^ (Figure S3, Table S1), indicating that it can be almost completely stripped after deposition. The results confirm that our experimental model is consistent with the proposed “escort effect” model in Scheme 1 and the configurations in the theoretical calculations.

**Figure 3:**
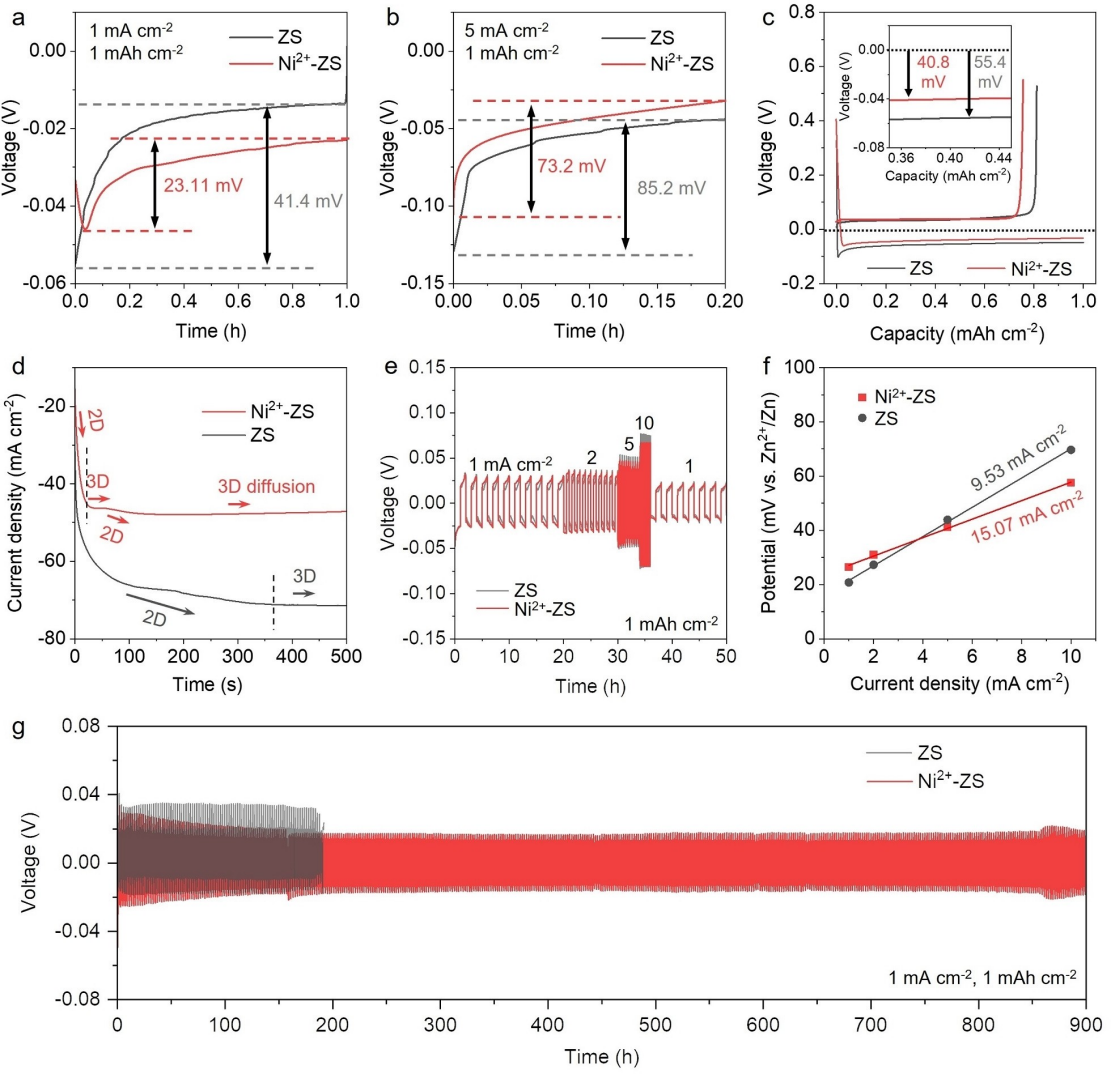
Electrochemical behaviours of Zn electrodes in ZS and Ni^2+^‐ZS electrolytes. The first discharge profiles of Zn||Zn symmetric cells at a) 1 mA cm^−2^ and b) 5 mA cm^−2^. c) Voltage profiles of Zn||Cu cells at 2 mA cm^−2^ (inset: magnified plating curves). d) Chronoamperograms (CAs) at an overpotential of −150 mV. e) Rate performances of Zn||Zn symmetric cells at different current densities with a fixed capacity of 1 mAh cm^−2^. f) Exchange current density from curves in e). g) Galvanostatic cycling curves of Zn||Zn symmetric cells at 1 mA cm^−2^ with a fixed capacity of 1 mAh cm^−2^.

Detailed electrochemical investigations were further implemented. As shown in Figure 3a,b, the nucleation overpotential of the Zn in cells with Ni^2+^‐ZS electrolyte was significantly smaller than the one in ZS electrolyte, both at low (1 mA cm^−2^) and high (5 mA cm^−2^) current densities. This demonstrates that Zn nucleates more easily on the Ni surface.^[15]^ Moreover, Figure 3c reveals that the polarization potential of Zn on Ni (40.8 mV) is smaller than that of Zn on Zn (55.4 mV), indicating that the growth process of Zn on Ni is also facilitated.^[16]^ We additionally tested the electrochemical behaviour of the Zn||Zn symmetric cells in the electrolytes with Cr^3+^ and Co^2+^ additives (denoted as Cr^3+^‐ZS and Co^2+^‐ZS), respectively. Note that both Cr and Co have higher work function and reduction potential than Zn (Table S2), which complies with the “escort effect”. As seen in Figure S4, the overpotential for Zn nucleation and growth in cells with Cr^3+^‐ZS and Co^2+^‐ZS electrolytes is smaller than that with the ZS electrolyte, indicating the universality of the “escort effect”.

Furthermore, chronoamperometry (CA) test was applied to investigate the behaviour of zinc deposition. At an overpotential of −150 mV, the current with ZS electrolyte did not stabilize (all nucleation sites being activated) until 200 s (Figure 3d); prior to this, it underwent a long rampant two‐dimensional (2D) zinc diffusion process in order to reduce the surface energy, during which Zn^2+^ tended to accumulate at the tip and generate dendrites.^[17]^ This contrast sample agrees with the literature.^[18]^ As a comparison, there are two interesting phenomena in cells with Ni^2+^‐ZS electrolytes. Firstly, the current reaches a steady state after only 30 s, and this current value is much smaller than the one in the ZS electrolyte, indicating that the active nucleation sites of the cell with Ni^2+^‐ZS electrolyte are much less than that with the ZS electrolyte. This may be related to the rapid nucleation of Zn on Ni, leading to a constrained 2D diffusion of Zn^2+^ and a reduction of Zn dendrites, thus reducing the surface area for nucleation. Another feature is that it anomalously undergoes two 2D‐3D diffusion processes in the Ni^2+^‐ZS CA curves, which corresponds to the nucleation, growth of Ni, and subsequent nucleation and growth of Zn, respectively. The alleviated concentration polarization during the Zn deposition and the uniform distribution of Zn^2+^ near the surface was also verified by the cyclic voltammetry (CV) test of Zn||Ti cells in the Ni^2+^‐ZS electrolyte (Figure S5). To evaluate the deposition kinetics more elaborately, the exchange current density associated with Zn deposition was calculated via Equation 1, ^[19]^

(1) $$i=i_{0}\frac{F}{RT}\frac{\eta }{2}$$

where *i* is the current density, *i_0_* is the exchange current density, *η* is the total overpotential, F is the Faraday constant, R is the gas constant, and *T* is the absolute temperature. As shown in Figure 3e,f, the Ni^2+^‐ZS has a higher value of exchange current density.

Generally, higher values of exchange current density indicate more favourable kinetics of metallic Zn deposition. These results indicate that the Ni^2+^‐contained electrolyte promotes the deposition of Zn in terms of both kinetics and thermodynamics. Moreover, the lower activation energy in the Ni^2+^‐ZS electrolyte suggests that the desolvation of Zn^2+^ is more facile than in the ZS electrolyte (Figure S6). Therefore, the performances of Zn||Zn symmetric cells with the Ni^2+^‐ZS electrolyte were enhanced, showing a long cycle life of more than 900 h at 1 mA cm^−2^ compared to less than 200 h for ZS electrolyte (Figure 3g). The Ni^2+^ additive also enabled Zn||Zn symmetric cells to operate for more than 1200 h at a high current density of 5 mA cm^−2^ (Figure S7) and to operate steadily at high capacities (Figure S8).

**Figure 4:**
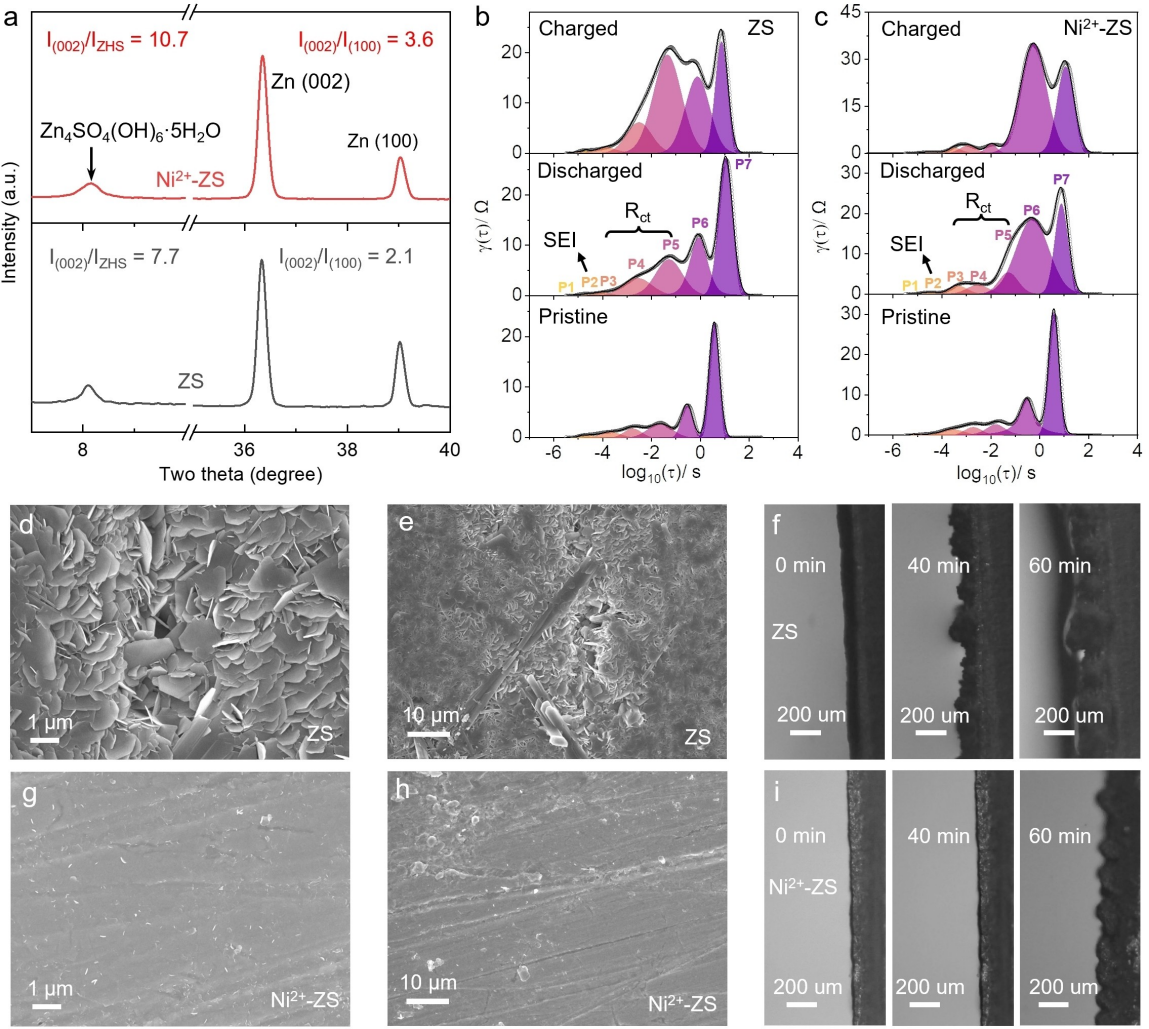
Material characterizations of the Zn electrode with ZS and Ni^2+^‐ZS electrolytes. a) GIXRD patterns of the Zn foil from Zn||Zn symmetric cells after 1 h of plating and 1 h of stripping at 1 mA cm^−2^. The distribution of relaxation times (DRT) plot of the in situ electrochemical impedance spectroscopy (EIS) data shown in Figure S9, attributed to b) ZS and c) Ni^2+^‐ZS. The P3‐P5 peaks are attributed to the interfacial charge transfer impedance. The SEM images of the Zn foils cycled with d), e) ZS electrolyte and g), h) Ni^2+^‐ZS electrolyte after 1 h plating and 1 h stripping at 1 mA cm^−2^. In situ optical microscopy images of Zn plating behaviours on a Zn substrate at 1 mA cm^−2^ with f) ZS and i) Ni^2+^‐ZS electrolytes.

In addition to the electrochemical study, we studied the Zn deposition process by spectroscopies and electron microscopy. First, the Zn foils after cycling were characterized by grazing incidence X‐ray diffraction (GIXRD), a powerful diagnose for investigating the texturing and orientation anisotropy of thin films.^[20]^ As shown in Figure 4a, after plating/stripping of certain amount of Zn at 1 mA cm^−2^, the intensity ratio of Zn(002) to Zn(100) is 3.6 for the Zn anode in the Ni^2+^‐ZS electrolyte and 2.1 for the one in the ZS electrolyte, indicating the enhanced Zn(002) plane in the Ni^2+^‐contained system. This may stem from the fact that the UPD of Zn on Ni prefers horizontal and continuous growth, which contributes to the tight deposition of Zn and reduces dendrites.[^3^, ^21^] Another phenomenon is that the intensity of Zn_4_SO_4_(OH)_6_⋅5H_2_O (ZHS) in the Ni^2+^‐ZS electrolyte is lower than that in the ZS electrolyte, as shown by I_(002)_/I_ZHS_ values, i.e. 10.7 for that in Ni^2+^‐ZS electrolyte and 7.7 for the one in ZS electrolyte. Note that this ZHS is caused by the local increased pH generated by the HER, a typical side reaction during Zn plating/stripping. In addition, the pH increase in Ni^2+^‐ZS electrolyte is less than that in ZS electrolyte (Table S3), which can be attributed to the migrated HER in the Ni^2+^‐ZS system. These phenomena are consistent with the behaviours mentioned above of Ni promoting Zn deposition, which leads to the deposition of Zn taking electrons from the HER. For a more accurate study, we recorded in situ EIS spectra of the two systems with different electrolytes (Figure S9) and performed DRT analysis of the corresponding EIS curves (Figure 4b,c). The DRT method is a more authentic way to analyse the changes at the electrode interface.^[22]^ We calibrated the peaks with different time constants. Among them, P3–P5 can be attributed to the peaks of interfacial charge transfer impedance (Table S4). It can be clearly seen that the interfacial charge transfer impedance in the ZS electrolyte increases significantly after the first cycle of plating/stripping, while that in the Ni^2+^‐ZS electrolyte remains relatively constant, corresponding to a significant decrease of the ZHS by‐product. Additional SEM and in situ optical microscopy measurements delivered intuitive results. It can be seen that a large number of small fragments of Zn dendrites/ZHS were generated on the surface of the Zn foil after deposition/stripping in the ZS electrolyte (Figure 4d,e), while only a few tiny clusters were generated in the Ni^2+^‐ZS electrolyte (Figure 4g,h), showing a positive effect on the uniform Zn deposition. Similar smooth surfaces of the Zn foil were also observed after cycling in Co^2+^‐ZS and Cr^3+^‐ZS electrolytes (Figure S10). We can also see from the optical microscopy results that only a few uniform protrusions are present on the surface of the Zn anodes after 60 min plating in the Ni^2+^‐ZS electrolyte, while the surface of the Zn foil after cycling in the ZS electrolyte has been corroded with a large number of Zn dendrites (Figure 4f, i). All these results demonstrate that the “escort effect” of Ni additives can effectively promote the uniform deposition of Zn and greatly alleviate the side reactions. Meanwhile, the “escort effect” of Ni^2+^ showed improved cycling stability of Zn full cells using commercial V_2_O_5_ and commercial MnO_2_ cathodes (Figure S11).

## Conclusion

In summary, we proposed an “escort effect” of electrolyte additives based on optimizing interfacial electrochemistry, namely, using metal ions with higher work function and deposition potential than Zn as electrolyte additives, which can deposit in advance and direct subsequent Zn deposition, and dissolve after Zn stripping. When using Ni^2+^ additives, through theoretical calculations and experimental characterizations including depth‐profile XPS and UPS, GIXRD, and DRT analysis towards in situ EIS spectra, we verified that the UPD of Zn on Ni enables the uniform Zn deposition, suppresses the side reaction, stabilizes the interfacial charge transfer impedance, and achieves a reversible Zn metal anode at a low current density of 1 mA cm^−2^. Based on Cr^3+^ and Co^2+^ additives, the generality of the “escort effect” was verified. The idea of “escort effect” will inspire a new series of electrolyte additive strategies to optimize metal deposition and also inspire other metal battery studies.

## Conflict of interest

The authors declare no conflict of interest.

1

## Supporting information

As a service to our authors and readers, this journal provides supporting information supplied by the authors. Such materials are peer reviewed and may be re‐organized for online delivery, but are not copy‐edited or typeset. Technical support issues arising from supporting information (other than missing files) should be addressed to the authors.

Supporting Information

## Data Availability

The data that support the findings of this study are available from the corresponding author upon reasonable request.
