# Supplementary material for: Reversible Zn Metal Anodes Enabled by Trace Amounts of Underpotential Deposition Initiators
Source: Angew Chem Int Ed Engl. 2023 Mar 24;62(18):e202301192. doi: 10.1002/anie.202301192 (PMC10946846; doi:10.1002/anie.202301192)
Supplement: Supplementary file 1 — Supporting Information [file ANIE-62-0-s001.pdf]

Angewandte Chemie
Eine Zeitschrift der Gesellschaft Deutscher Chemiker

Supporting Information

Reversible Zn Metal Anodes Enabled by Trace Amounts of Underpotential Deposition Initiators

Y. Dai, C. Zhang, W. Zhang, L. Cui, C. Ye, X. Hong, J. Li, R. Chen, W. Zong, X. Gao, J. Zhu, P. Jiang, Q. An, D. J. L. Brett, I. P. Parkin, G. He, L. Mai**

Experimental Procedures

1. Electrolyte and electrode preparation

The bare 2 M $ZnSO_4$ electrolyte (ZS) was prepared by dissolving 0.1 mol $ZnSO_4 \cdot 7H_2O$ into 50 mL deionized water under vigorous magnetic stirring for 20 min. The 2 M ZnSO4 + 0.004 M $NiSO_4$ (Ni^{2+}-ZS) were then prepared by doing the same procedure followed by adding 0.0002 mol $NiSO_4 \cdot 6H_2O$ into the ZS. The V_2O_5 and MnO_2 cathodes were prepared by the same method; specifically, the cathode was composed of 70 wt.% commercial powder of active material (V_2O_5 and MnO_2), 20 wt.% acetylene black, and 10 wt.% poly(vinylidene fluoride) (average Mw ~534000) with N-methyl-2-pyrrolidine as the solvent. Hydrophilic type carbon paper was served as the current collector.

2. Material characterizations

Scanning electron microscope (SEM) was conducted on JEOL-JSM-6700F (Voltage: 5 kV, emission current: 110.8 μA). The *in-situ* optical microscope was performed on a VisiScope® BL254 T1 (VWR) instrument with a purpose-designed electrolytic cell in a symmetric configuration of Zn||Zn. The X-ray diffraction (XRD) patterns of Zn foils were obtained on a PANalytical Empyrean device (Cu Kα radiation; 40 kV, 40 mA) in the range of 5° to 80° with a step size of 0.05° and a scan rate of 0.1° s^{-1}. The chemical states and work function information and their evolution at different discharge states were investigated by X-ray photoelectron spectroscopy and ultraviolet photoelectron spectroscopy (XPS and UPS, Kratos Axis SUPRA). Argon ion etching of XPS and UPS was carried out using a rate of 10 nm min^{-1}.

3. Electrochemical measurements

The Zn||Zn symmetric cells, Zn||Cu cells, Zn||MnO_2 full cell, and Zn||V_2O_5 full cells were assembled to evaluate the electrochemical performances. They were based on CR2032 coin cell in Neware battery test system (Shenzhen, China). The amounts of electrolytes for each cell were controlled as 120 μL. The thickness of Zn anode is 70 μm. A Biologic VMP-3 electrochemical workstation was used to conduct *in-situ* electrochemical impedance spectroscopy (EIS), chronoamperogram (CA) of Zn||Zn symmetric cells at an overpotential of -150 mV, linear polarization curves at 10 mV s^{-1} (with Zn foil as the working electrode, Pt as the counter electrode and Ag/AgCl as the reference electrode), and LSV curves based on Zn||Ti half cells at 5 mV s^{-1}. The *in-situ* EIS test was set to first discharge for 1 h and then charge for 1 h at a current density of 1 mA cm^{-2}. The pH evolution of ZS and Ni^{2+}-ZS electrolytes after cycling in Zn||Zn cells was performed based on a two-electrode system in a 15 mL beaker, where the distance between the two electrodes was ca. 2 cm.

4. Computational methods and models

All the DFT simulations in the work were performed within a periodic model by the Vienna ab initio simulation program (VASP)[1]. The generalized gradient approximation (GGA) was used with the Perdew-Burke-Ernzerhof (PBE) exchange-correlation functional[2]. The projector-augmented wave (PAW) method was utilized to describe the interactions of the electron-nucleus[3], and the cut-off energy for the plane-wave basis set was 450 eV. Brillouin zone integration was set as 2×2×1 Monkhorst-Pack k-point mesh for systems with surfaces. All the adsorption geometries were optimized using a force-based conjugate gradient algorithm. The nudged elastic band (NEB) method was applied to find the transition state for the migration barrier[4]. In all the calculations, SCF convergence, max force, stress, displacement is set to 550 eV, $2.0 \times e{-5}$ eV/atom, 0.05 eV/Å, 0.1 GPa, and 0.002 Å, respectively.

5. Detail of the distribution of relaxation times (DRT) analysis of EIS spectra

DRT analysis was conducted *via* DRTtools (https://github.com/ciuccislab/DRTtools) supported by Francesco Ciucci. The hierarchical Bayesian approach was selected to analyze original EIS data coupled with Gaussian method of discretization[5].

Results and Discussion

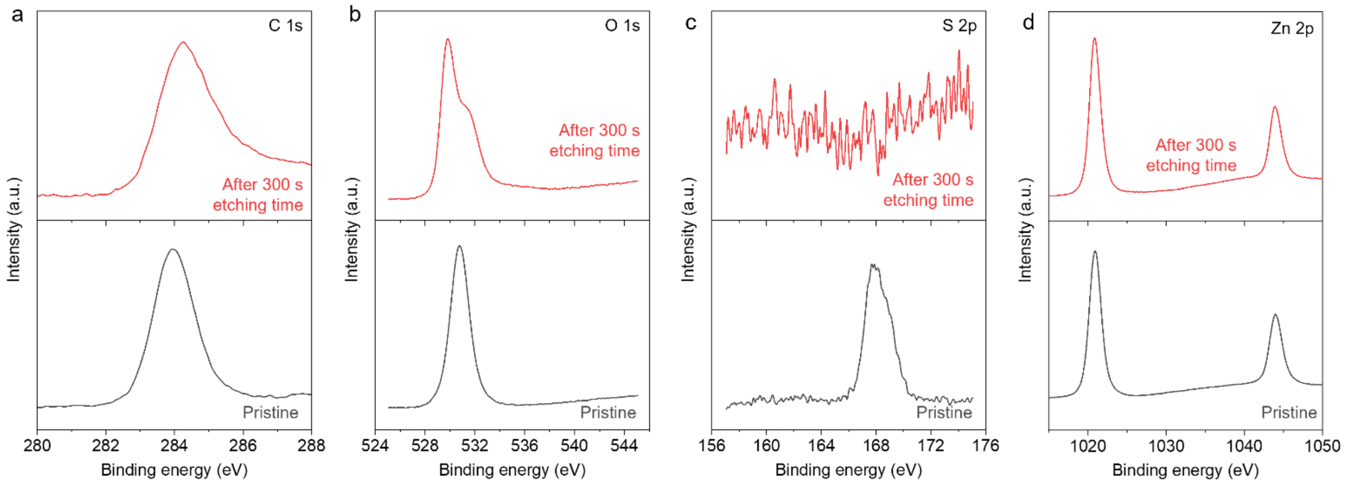

Figure S1. XPS spectra of a) C 1s, b) O 1s, c) S 2p, and d) Zn 2p of the Zn foil after 1 h of plating at 1 mA cm^{-2} in a Zn||Zn symmetric cell with a Ni^{2+}-ZS electrolyte. The curves include pristine states and states after 300 s etching time.

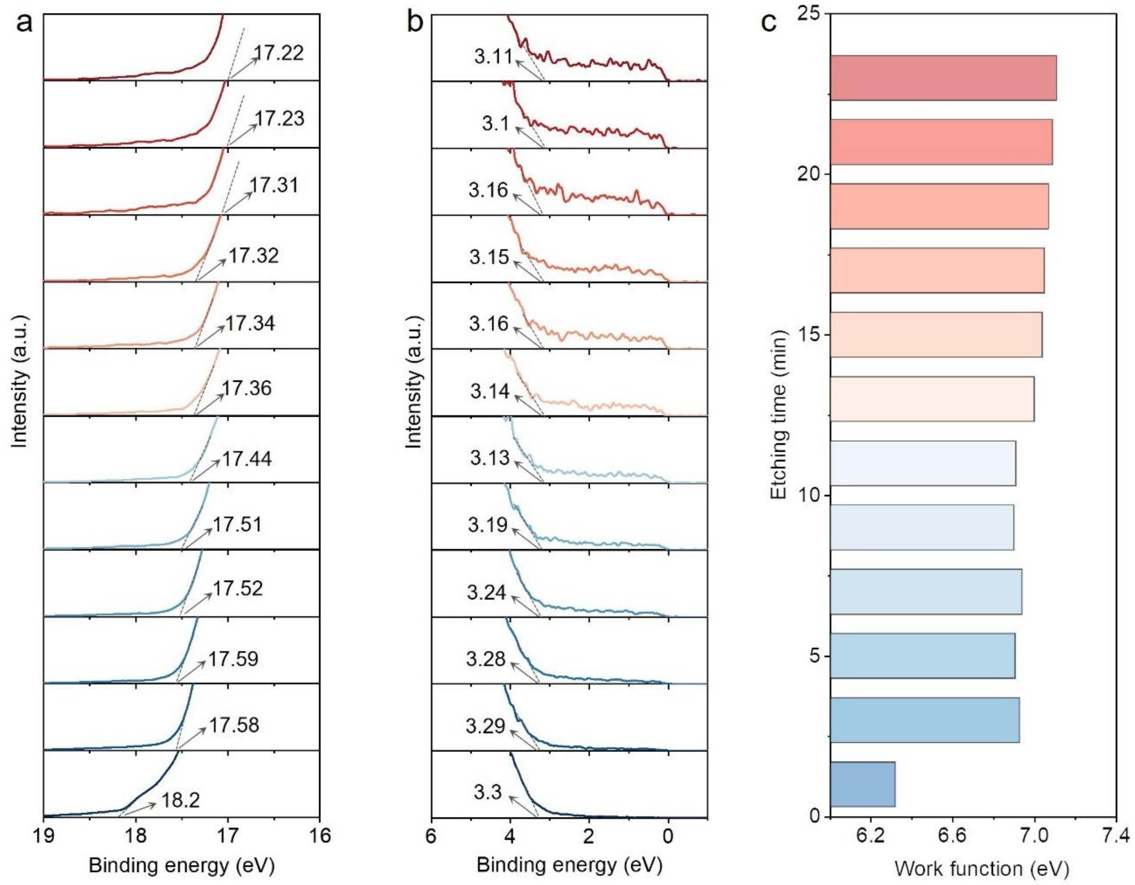

Figure S2. Detailed investigation of the Zn surface after 4 min plating at 1 mA cm^{-2} with ZS electrolytes, including a,b) UPS spectra at states after etching for 1 min, 3 min, 5 min, 7 min, 9 min, 11 min, 13 min, 15 min, 17 min, 19 min, 21 min, and 23 min from bottom to top, and c) Calculated work function at corresponding states in a) and b).

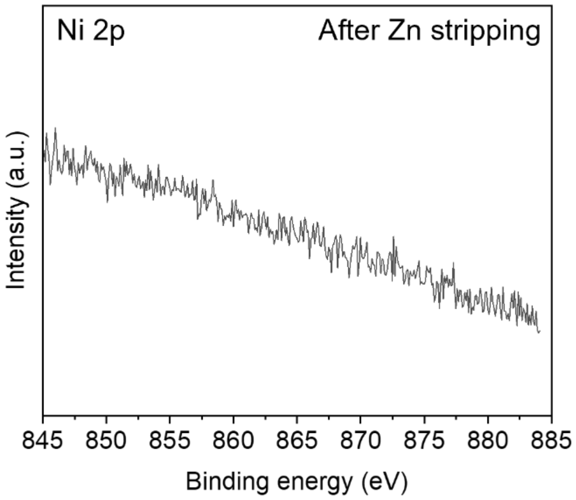

Figure S3. Ni 2p XPS spectra of the Zn electrode in a Zn||Zn symmetric cell with a Ni^{2+}-ZS electrolyte after 1 h plating and 1 h stripping at 1 mA cm^{-2}.

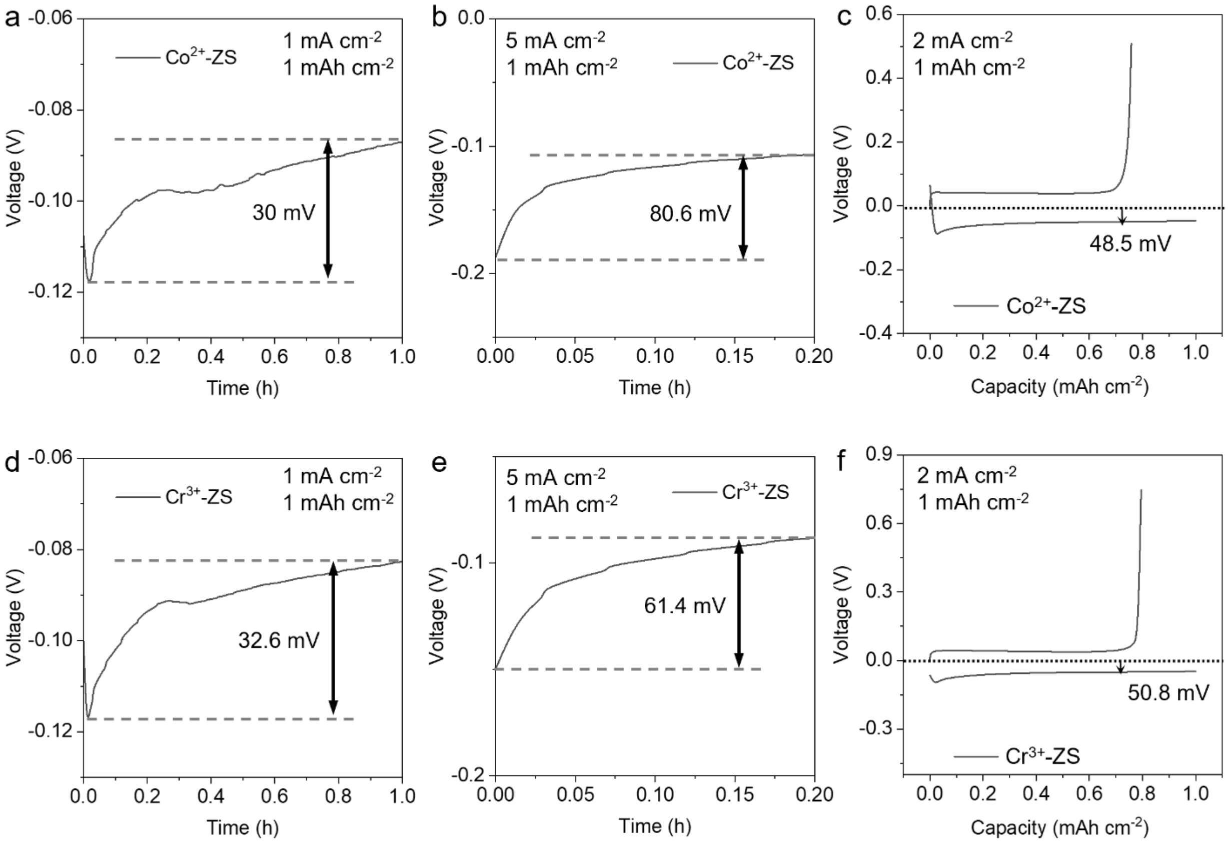

Figure S4. Electrochemical behaviours for Zn electrodes in Co^{2+}-ZS and Cr^{3+}-ZS electrolytes. The first discharge profiles of Zn||Zn symmetric cells at a) 1 mA cm^{-2} and b) 5 mA cm^{-2}. c) Voltage profiles of Zn||Cu cells at 2 mA cm^{-2} (inset: magnified plating curves).

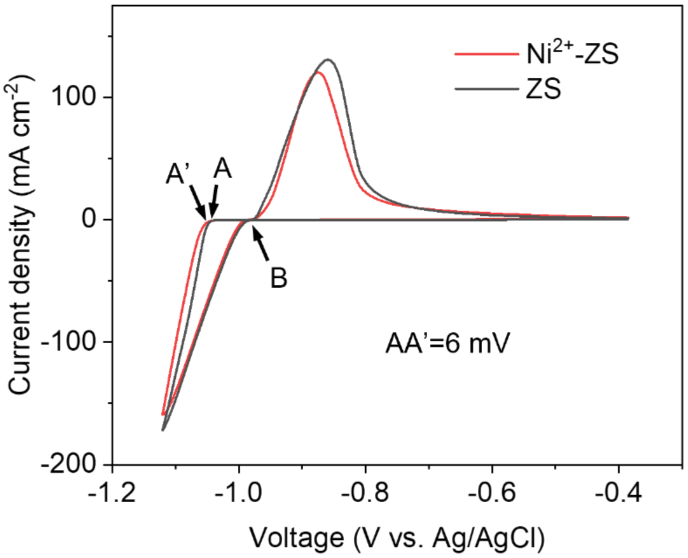

Figure S5. Cyclic voltammograms (CV) for Zn nucleation on bare Ti foil in Ni^{2+}-ZS and ZS electrolytes under the scan rate of 10 mV s^{-1}. The reduction potential changed from A to A' with the addition of Ni^{2+}, indicating that the rapid growth sites of zinc were blocked and the concentration polarization during deposition was alleviated. Thus, the distribution of Zn^{2+} was more uniform in Ni^{2+}-ZS than in ZS.

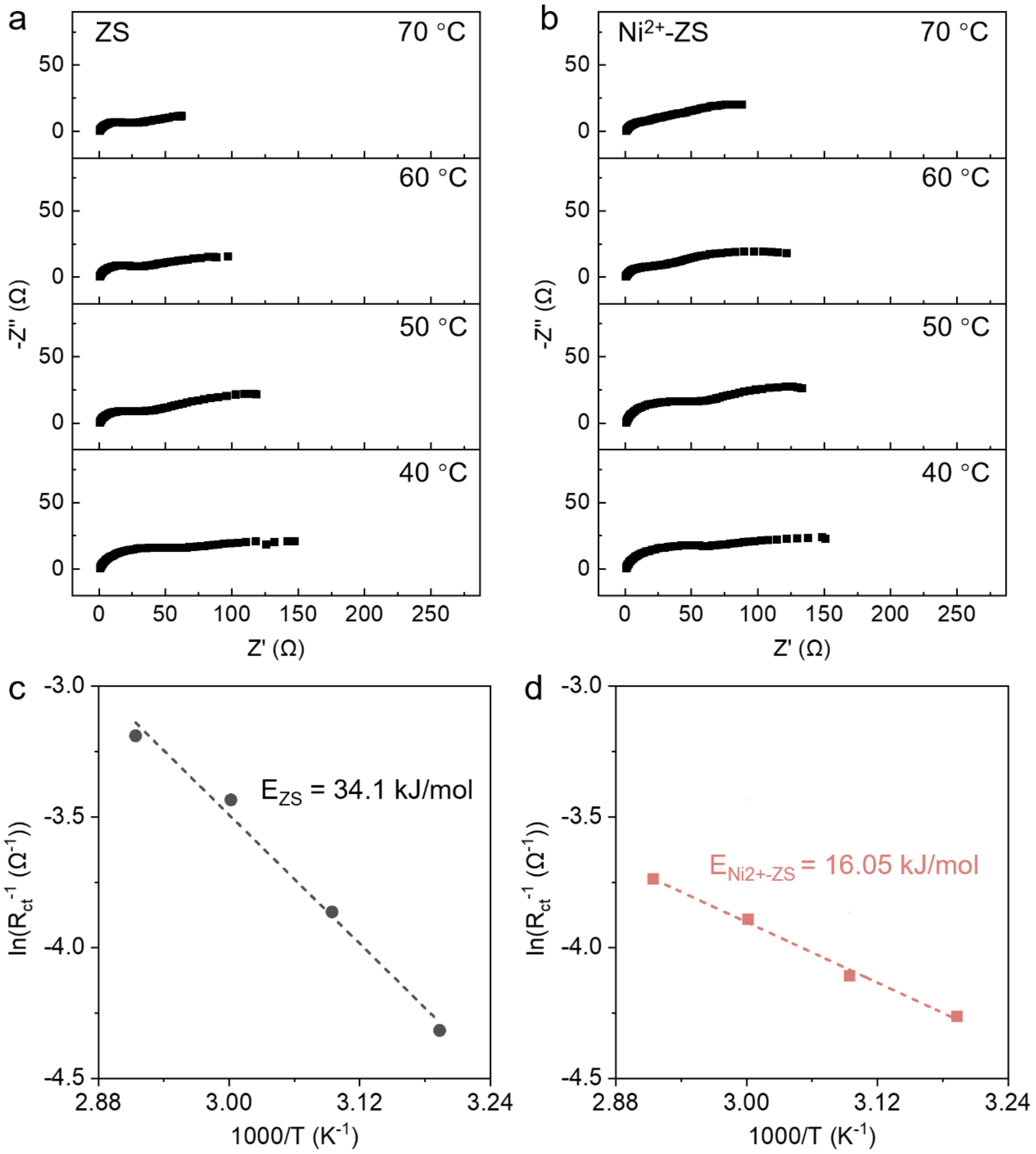

Figure S6. a,b) Nyquist plots of Zn||Zn symmetric cells in ZS and Ni^{2+}-ZS electrolytes recorded at different temperatures (from 40 °C to 70 °C). c,d) Arrhenius plots of inverse R_{ct} (R_{ct}^{-1}) values at different temperatures (from 40 °C to 70 °C).

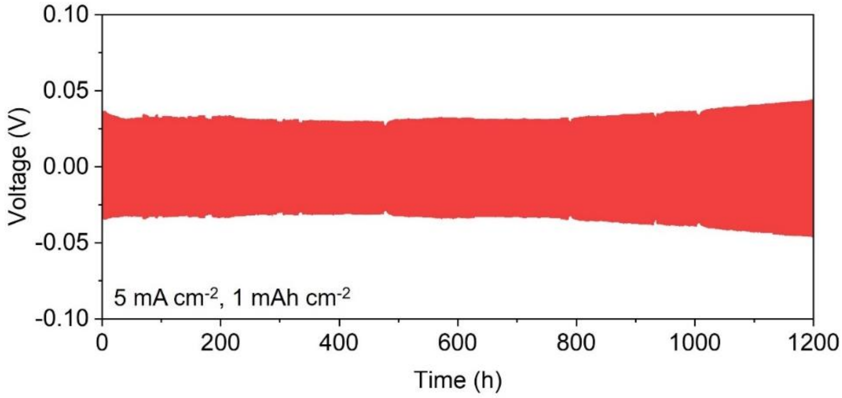

Figure S7. Galvanostatic cycling curves of Zn||Zn symmetric cells with Ni^{2+}-ZS electrolyte at 1 mA cm^{-2} with a fixed capacity of 1 mAh cm^{-2}.

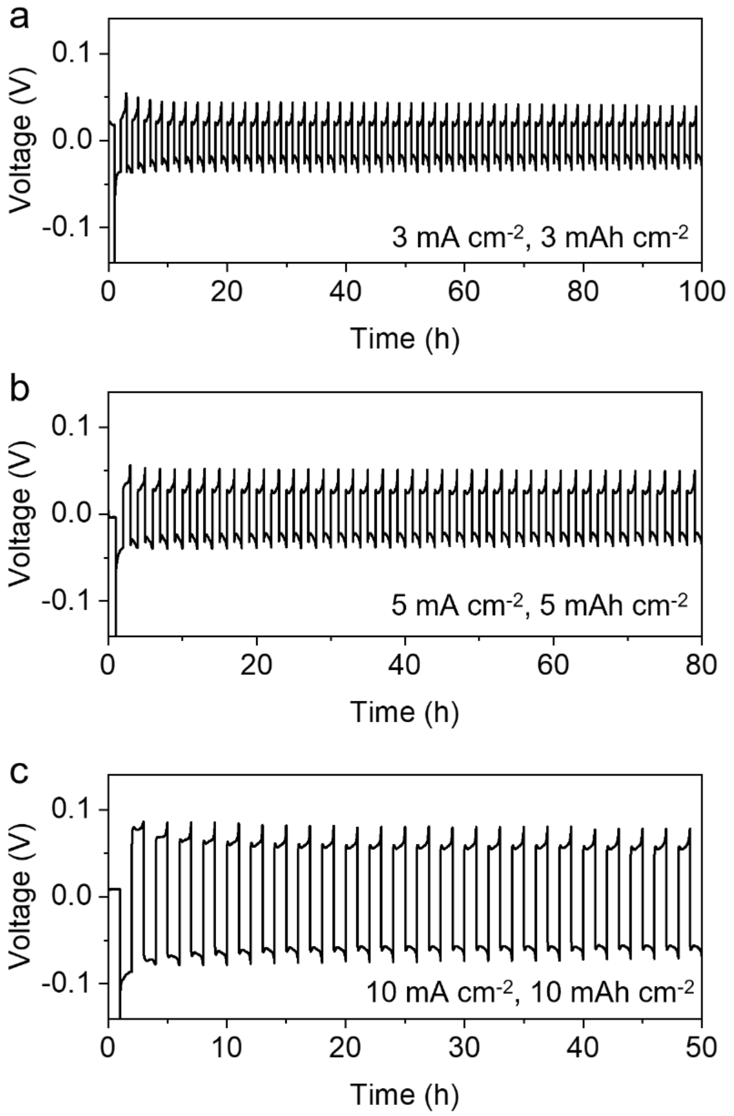

Figure S8. Galvanostatic cycling curves of Zn||Zn symmetric cells with Ni^{2+}-ZS electrolyte at 3 mA cm^{-2}, 5 mA cm^{-2}, 10 mA cm^{-2} with a charge/discharge time of 1 h.

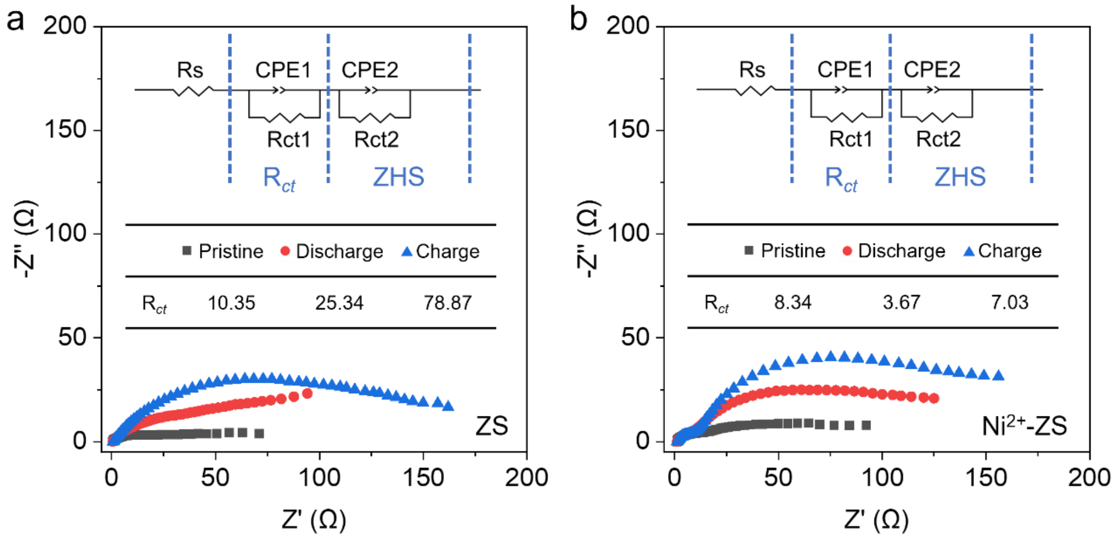

Figure S9. Nyquist plots of Zn||Zn symmetric cells with ZS and Ni^{2+}-ZS electrolytes, which were recorded at the pristine, discharged and charged states from *in-situ* EIS tests. The R_{ct} values obtained from the "equivalent circuit" method also indicate that the interfacial charge transfer resistance varies considerably in the ZS electrolyte, while it remains stable in the Ni^{2+}-ZS electrolyte.

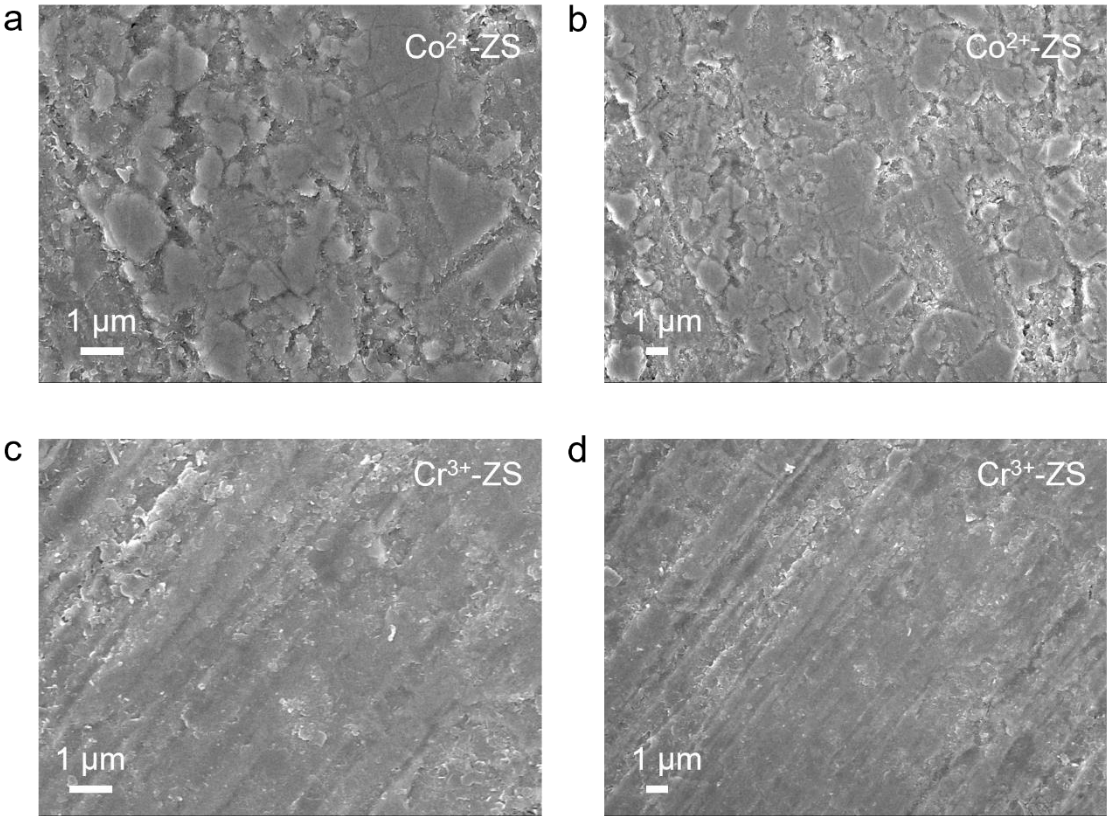

Figure S10. SEM images of the Zn foils cycled with a,b) Co^{2+}-ZS electrolyte and c,d) Cr^{3+}-ZS electrolyte after 1 h plating and 1 h stripping at 1 mA cm^{-2}.

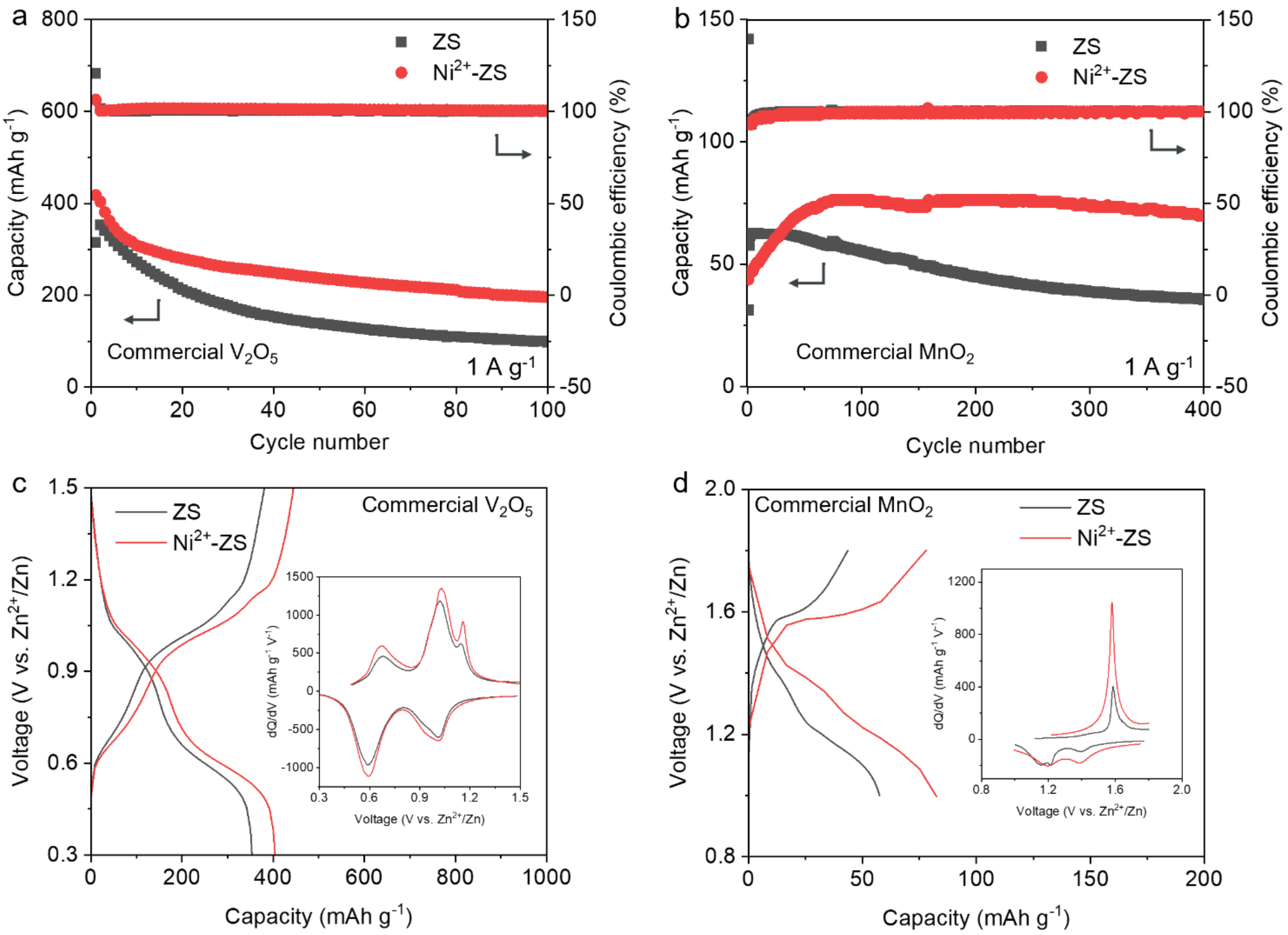

Figure S11. a,b) Galvanostatic cycling performances of Zn||V_2O_5 full cell and Zn||MnO_2 full cell, respectively, at a current density of 1 A g^{-1}. c,d) Galvanostatic charge-discharge curves of Zn||V_2O_5 full cell and Zn||MnO_2 full cell, respectively. The insets in c) and d) are corresponding dQ/dV curves.

Table S1. Energy-dispersive X-ray spectroscopy (EDX) results of the surface of the Zn foils in the discharged and charged states with the Ni^{2+}-ZS electrolyte.

	At%	
	Discharged	Charged
Zn	57	80
O	9.3	20
Ni	0.3	0
S	0.2	0

Table S2. Information of the work function and reduction potential of the metals.

Elements	Reduction potential (V vs. SHE)	Work function (eV)
Zn	-0.76	4.33
Ni	-0.257	5.15
Cr	-0.74	4.5
Co	-0.28	5

Table S3. pH evolution of the electrolytes before and after cycling in a Zn||Zn symmetric cell.

	Before cycling	After cycling
ZS	3.78	5.48
Ni^{2+}-ZS	3.80	5.38

Table S4. Summary of application of DRT peaks calculated for a Zn||Zn symmetric cell[6].

DRT Peak	Approximate time constant, τ	Assignment
P1	13.2-20 µs	Double-layer relaxation
P2	21.9-44.5 µs	Solid-electrolyte interphase
P3	0.11-0.5 ms	Positive electrode charge transfer
P4	1.2-3.3 ms	Positive electrode charge transfer
P5	11-56 ms	Positive electrode charge transfer
P6	0.3-0.91 s	Diffusion
P7	3.95-12.02 s	

Supplementary notes

1. Supplementary elucidation according to the Pourbaix diagrams. It can be observed in Figure S12 that the Ni^{2+}/Ni redox potential is higher than Zn^{2+}/Zn, which ensures that Ni deposition occurs in advance of Zn deposition, thus following the "escorting effect" in the main text.

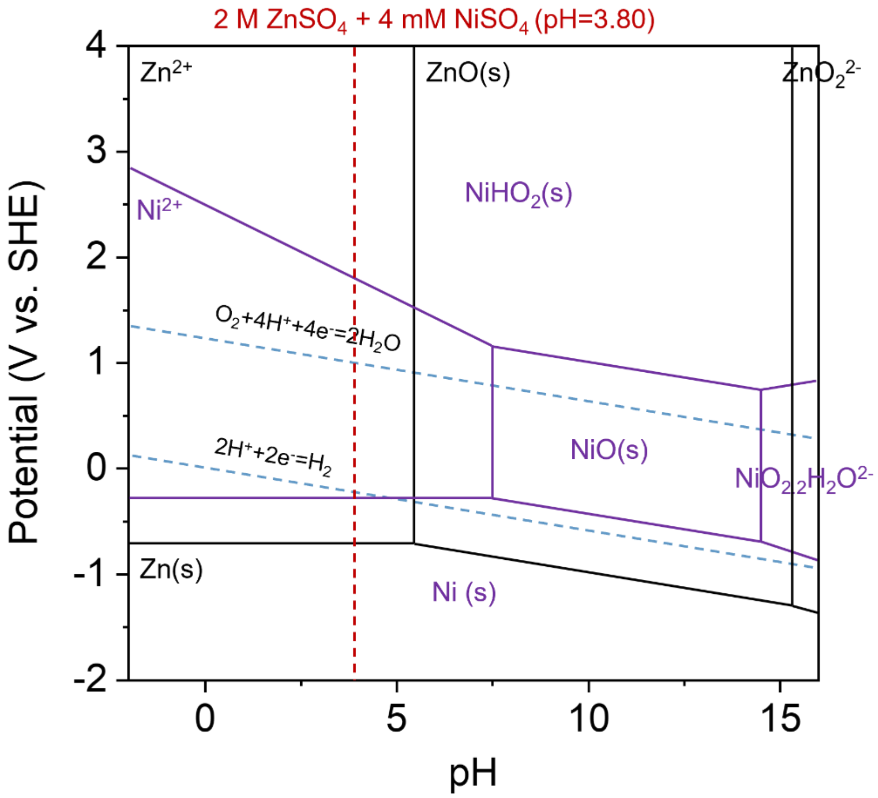

Figure S12. Zn-H_2O Pourbaix diagram and Ni-H_2O Pourbaix diagram. Both diagrams were extracted from the Materials Project.

2. Investigation of the effect of the introducing of Ni on HER reaction. According to Figure S13a, the HER onset potential is similar for ZS and Ni^{2+}-ZS electrolytes. Meanwhile, the corrosion current density in the Ni^{2+}-ZS electrolyte is smaller than that in the ZS electrolyte (Figure S13b). Therefore, the introduction of Ni does not accelerate HER, which is similar to the previous report[7].

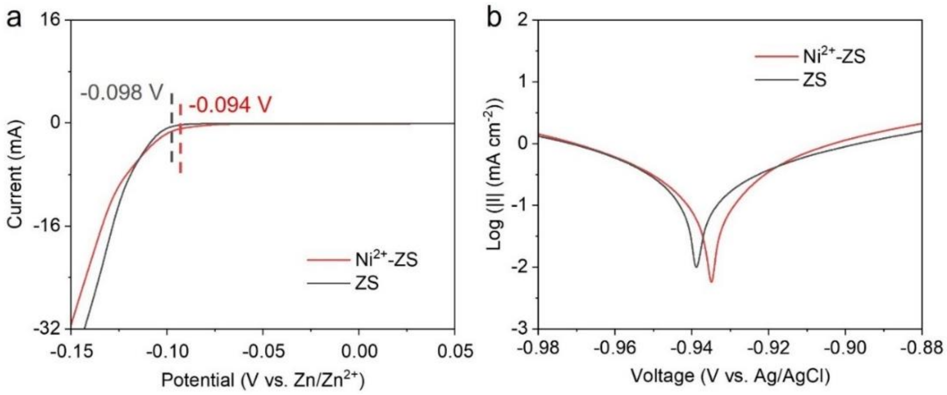

Figure S13. Electrochemical behaviours in ZS and Ni^{2+}-ZS electrolytes. a) LSV curves presenting the hydrogen evolution reaction (HER) region. b) Linear polarization curves of Zn anodes.

References

[1] a) G. Kresse, J. Furthmuller, *Phys. Rev. B. Condens. Matter.* **1996**, *54*, 11169-11186; b) G. Kresse, J. Furthmuller, *Comput. Mater. Sci.* **1996**, *6*, 15-50.
[2] J. P. Perdew, K. Burke, M. Ernzerhof, *Phys. Rev. Lett.* **1996**, *77*, 3865-3868.
[3] D. Vanderbilt, *Phys. Rev. B. Condens. Matter.* **1990**, *41*, 7892-7895.
[4] a) T. A. Halgren, W. N. Lipscomb, *Chem. Phys. Lett.* **1977**, *49*, 225-232; b) K. Burke, *J. Chem. Phys.* **2012**, *136*, 150901; c) K. Mathew, R. Sundararaman, K. Letchworth-Weaver, T. A. Arias, R. G. Hennig, *J. Chem. Phys.* **2014**, *140*, 084106.
[5] T. H. Wan, M. Saccoccio, C. Chen, F. Ciucci, *Electrochim. Acta* **2015**, *184*, 483-499.
[6] R. Soni, J. B. Robinson, P. R. Shearing, D. J. L. Brett, A. J. E. Rettie, T. S. Miller, *Energy Storage Mater.* **2022**, *51*, 97-107.
[7] Y. Gao, Q. Cao, J. Pu, X. Zhao, G. Fu, J. Chen, Y. Wang, C. Guan, *Adv. Mater.* **2022**, *35*, e2207573.

Author Contributions

L. Mai and G. He led the research work. Y. Dai and W. Zhang conceived and designed this work. C. Zhang carried out the DFT simulations. L. Cui conducted the UPS characterizations. X. Hong led the DRT analysis towards the *in-situ* EIS spectra. J. Li, C. Ye and P. Jiang conducted materials synthesis and electrochemical measurements, as well as participated in the writing of the draft. R. Chen, W. Zong, X. Gao, J. Zhu, Q. An, D. J.L. Brett, and I. P. Parkin participated in the analysis and discussion of the results from beginning to end. All authors discussed the results and commented on the manuscript.
